# Rapidly progressive subdural empyema secondary to acute pansinusitis complicated by superior sagittal sinus thrombosis in a young adult: a multidisciplinary case report

**DOI:** 10.25122/jml-2026-0043

**Published:** 2026-07

**Authors:** Roxana Nartea, Monica Hodor, Simona Vetu, Ilinca Pirciog

**Affiliations:** 1 Carol Davila University of Medicine and Pharmacy, Bucharest, Romania; 2 National Institute of Rehabilitation, Physical Medicine and Balneology, Bucharest, Romania; 3 Neurosurgery Department, Floreasca Emergency Hospital, Bucharest, Romania

**Keywords:** subdural empyema, pansinusitis, superior sagittal sinus thrombosis, *Prevotella oris*, neurosurgery, otorhinolaryngology, neurorehabilitation, CT, computed tomography, GCS, Glasgow Coma Scale, ICU, intensive care unit, MRV, magnetic resonance venography

## Abstract

Subdural empyema is a rare but life-threatening intracranial infection, most commonly arising as a complication of paranasal sinusitis. Early diagnosis and multidisciplinary treatment are essential to reduce morbidity and mortality. A 23-year-old previously healthy male developed a rapidly progressive subdural empyema associated with superior sagittal sinus thrombosis and severe sepsis. Clinical deterioration occurred within 24 hours of symptom onset. Cranial computed tomography revealed a left fronto-temporo-parietal subdural collection with mixed density and air inclusions, along with extensive pansinusitis. Emergency neurosurgical evacuation was followed by otorhinolaryngological surgical management. Broad-spectrum intravenous antibiotics and anticoagulation therapy were administered under intensive care monitoring. Microbiological analysis identified *Prevotella oris*. Postoperatively, the patient required structured neurorehabilitation for right-sided hemiparesis and mixed aphasia, achieving gradual functional recovery. Gradual neurological and functional improvement was observed during the follow-up period. This case highlights the importance of early diagnosis, prompt multidisciplinary management, and comprehensive rehabilitation in improving outcomes. Although a favorable recovery was achieved, conclusions regarding causality and treatment efficacy are limited by the single-case design.

## Introduction

Subdural empyema represents a neurosurgical emergency characterized by the accumulation of purulent material between the dura mater and arachnoid membrane [1]. Despite advances in imaging and antimicrobial therapy, it remains associated with significant morbidity and mortality [1-3]. Paranasal sinusitis represents the most common source of infection in adolescents and young adults, with intracranial spread occurring via valveless venous channels or direct extension. Historically, the incidence of intracranial complications of sinusitis has been estimated at 1–2%. However, recent evidence suggests a concerning increase in these complications in the post-COVID-19 era, potentially related to delayed presentations and altered healthcare access [4].

Intracranial spread may be further complicated by cerebral venous sinus thrombosis, including involvement of the superior sagittal sinus, which significantly worsens prognosis and requires complex multidisciplinary management [5,6].

This case report describes a rapidly progressive subdural empyema secondary to acute pansinusitis in a previously healthy young adult, complicated by superior sagittal sinus thrombosis. It also highlights the role of coordinated surgical, medical, and rehabilitation strategies.

## Case presentation

This case report was prepared in accordance with the CARE guidelines.

### Patient information

A 23-year-old man with no prior medical history presented with a sudden-onset frontal headache, neck stiffness, confusion, and lethargy. No history of trauma, chronic disease, or immunosuppression was noted.

### Clinical findings

At admission, the patient presented with:

- Temperature: 38.2°C
- Heart rate: 112 bpm
- Blood pressure: 128/74 mmHg
- Oxygen saturation: 95% on room air

Neurological examination revealed a Glasgow Coma Scale score of 9/15 and an inability to follow commands. Meningeal irritation was present (positive Brudzinski sign). Due to impaired consciousness, orotracheal intubation and admission to the intensive care unit were required.

### Timeline of events

**Table:** 

Date	Event
Jan 4, 2026	Onset of frontal headache, neck stiffness, and confusion
Jan 5, 2026	Neurological deterioration, intensive care unit (ICU) admission, CT scan
Jan 5, 2026	Emergency craniotomy and subdural evacuation
Jan 6–7, 2026	Ear, nose, and throat (ENT) sinus surgery
Jan 8–15, 2026	Intravenous (IV) antibiotics and anticoagulation, ICU care
Feb 12, 2026	Discharge from acute care, transfer to rehabilitation
Mar 4, 2026	Ongoing rehabilitation, partial functional recovery

### Diagnostic assessment

**Figure 1:**
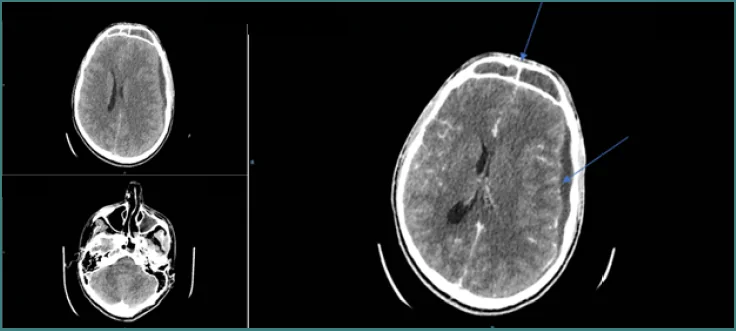
Initial cranial CT scan demonstrating a left fronto-temporo-parietal subdural collection (maximum thickness 13 mm) with mixed density and intralesional air bubbles, suggestive of anaerobic infection. An associated mass effect with mild midline shift is noted. Concomitant opacification of all paranasal sinuses is visible.

Initial cranial CT scan revealed a 13-mm left fronto-temporo-parietal subdural collection with mixed density and air inclusions, suggestive of anaerobic infection, associated with mild mass effect (Figure 1). Paranasal sinus CT demonstrated opacification of the frontal, maxillary, ethmoidal, and sphenoidal sinuses. Magnetic resonance venography confirmed superior sagittal sinus thrombosis.

**Figure 2:**
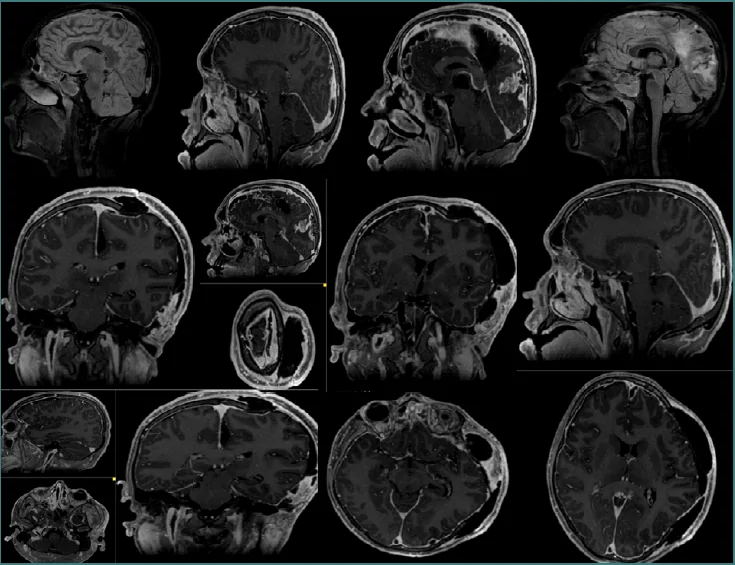
MR venography demonstrating superior sagittal sinus thrombosis with absence of normal flow-related signal within the superior sagittal sinus

Magnetic resonance venography confirmed superior sagittal sinus thrombosis (Figure 2).

Postoperative MRI demonstrated a left occipital abscess characterized by a ring-enhancing lesion with surrounding vasogenic edema. A follow-up CT scan identified a parafalcine subdural empyema without significant mass effect.

Laboratory findings included leukocytosis (18,500/mm^3^), elevated C-reactive protein (205 mg/L), and procalcitonin (5.2 ng/mL). Microbiological analysis of subdural pus identified *Prevotella oris*, while blood cultures were negative.

### Differential diagnosis

Based on the patient’s clinical presentation and neuroimaging findings, the differential diagnosis included subdural empyema, epidural abscess, brain abscess, bacterial meningitis, and complicated sinusitis.

### Therapeutic interventions

**Figure 3:**
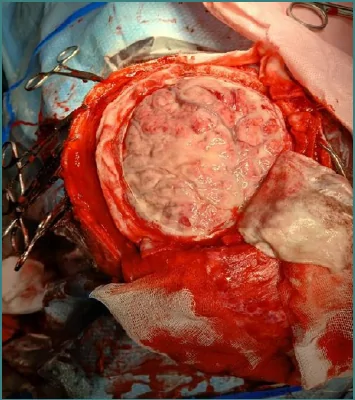
Intraoperative view during left fronto-temporo-parietal craniotomy showing evacuation of a purulent subdural collection

Emergency neurosurgical treatment was performed immediately after diagnosis. A left fronto-temporo-parietal craniotomy was undertaken under general anesthesia. Following dural opening, a thick purulent subdural collection was encountered and evacuated. Copious irrigation of the subdural space with sterile saline was performed until clear effluent was obtained. Purulent material samples were collected for microbiological analysis. The cerebral cortex appeared edematous but viable, without evidence of cortical abscess formation at the operative site. A subdural drain was left in place per institutional practice and was removed after satisfactory postoperative imaging (Figure 3). Due to the marked cerebral edema, the bone flap could not be replaced.

**Figure 4:**
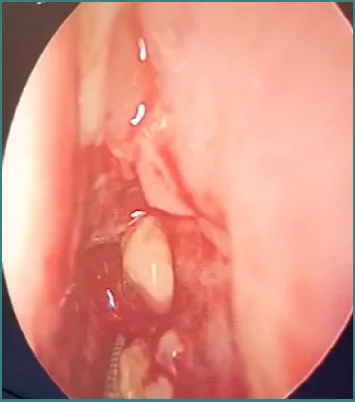
Endoscopic view of the paranasal sinuses demonstrating purulent secretions and inflamed mucosa consistent with acute pansinusitis

On postoperative day 2, the patient underwent ear, nose, and throat (ENT) surgery, including bilateral maxillary antrostomies, bilateral anterior and posterior ethmoidectomies, and bilateral Draf IIa frontal sinusotomies, resulting in the evacuation of copious purulent material. Teeth 1.6 and 2.6 were also extracted (Figure 4).

**Figure 5:**
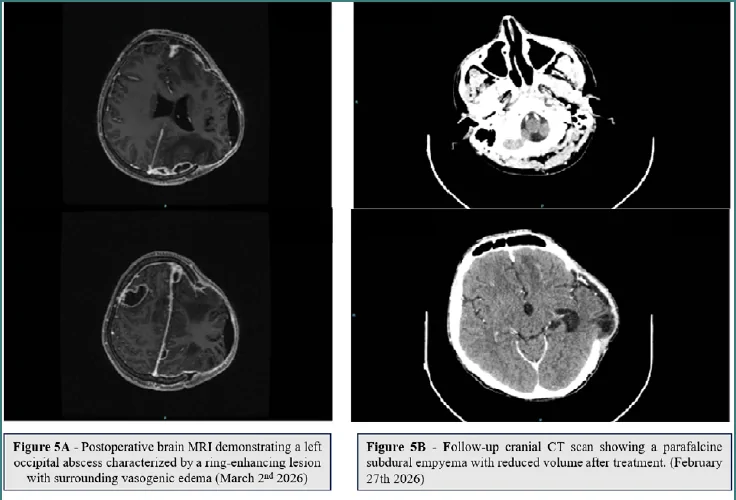
Postoperative neuroimaging demonstrating the evolution of intracranial infection following surgical treatment. A, Postoperative MRI of the brain demonstrating a ring-enhancing left occipital abscess with surrounding vasogenic edema (March 2, 2026). B, Follow-up cranial CT demonstrating a reduced parafalcine subdural empyema after treatment (February 27, 2026).

Medical management included broad-spectrum intravenous antibiotics, anticoagulation therapy for venous sinus thrombosis, and intensive care support.

The parafalcine empyema was managed conservatively with targeted antibiotic therapy, with subsequent radiological improvement.

### Outcome and follow-up

Postoperatively, the patient developed right-sided hemiparesis, mixed aphasia, and coordination deficits (Figure 5AB). Functional assessment at admission to the rehabilitation unit demonstrated severe impairment, with a Barthel Index (BI) score of 30/100.

Postoperative magnetic resonance imaging revealed a left occipital abscess, appearing as a ring-enhancing lesion with central diffusion restriction and surrounding vasogenic edema, consistent with a pyogenic process.

A control cranial CT scan demonstrated a parafalcine subdural empyema. Given its limited size and absence of significant mass effect, a conservative approach was adopted. The lesion showed progressive radiological improvement under targeted antibiotic therapy.

A structured multidisciplinary rehabilitation program was initiated, including physiotherapy and occupational therapy, resulting in gradual improvement of motor function and increased independence in daily activities. A delayed cranioplasty is planned.

## Discussion

Subdural empyema remains a rare but highly aggressive intracranial infection, most commonly arising as a complication of paranasal sinusitis, particularly in young, otherwise healthy individuals. Despite advances in neuroimaging and antimicrobial therapy, the condition continues to be associated with substantial morbidity and non-negligible mortality, especially when diagnosis and intervention are delayed [1,5]. The present case illustrates a fulminant clinical course, emphasizing the importance of early recognition and rapid multidisciplinary management.

A notable feature of this case is the hyperacute neurological deterioration occurring within 24 hours of symptom onset, whereas a more subacute progression is typically reported in the literature. One possible explanation for this rapid progression is the extensive involvement of multiple sinus compartments together with a virulent anaerobic infection [7–9]. The presence of intralesional air on computed tomography suggested an anaerobic etiology, which was subsequently confirmed microbiologically. The identification of *Prevotella oris* raises the possibility of a mixed sinonasal and odontogenic source. Although the extraction of teeth 1.6 and 2.6 raised the possibility of an odontogenic contribution, the precise origin of the infection could not be definitively established. Intracranial dissemination likely occurred via valveless venous channels or direct extension, as previously described [9–12].

The coexistence of superior sagittal sinus thrombosis represents a severe complication associated with worse neurological outcomes. Venous imaging played an important role in establishing the diagnosis and guiding management. The presence of intralesional air on computed tomography suggested an anaerobic etiology, which was subsequently confirmed microbiologically.

Recent literature has reported an increased incidence of intracranial complications of sinusitis in the post-COVID-19 era, possibly related to delayed presentations and changes in healthcare access. This trend highlights the need for heightened clinical vigilance in patients presenting with severe headache, fever, and altered mental status [4,13].

From a microbiological perspective, anaerobic bacteria are frequently implicated in sinonasal subdural empyema, although *Prevotella* species are less commonly reported. This finding supports the inclusion of adequate anaerobic coverage in empirical antimicrobial regimens, followed by targeted therapy based on culture results [7,8,14].

The management approach adopted in this case aligns with current evidence-based recommendations emphasizing urgent surgical drainage combined with eradication of the primary infectious focus. The staged multidisciplinary strategy achieved source control in this patient; however, the impact on recurrence risk cannot be determined from a single case. In addition, the use of anticoagulation therapy in the presence of venous sinus thrombosis reflects current practice, although it remains a complex clinical decision requiring careful risk–benefit assessment, particularly in the postoperative setting [10,14].

An important aspect of this case is the role of early, structured neurorehabilitation in functional recovery. While the acute management of subdural empyema is well established, data on long-term outcomes and rehabilitation strategies remain limited. The observed clinical improvement occurred following multidisciplinary rehabilitation; however, the contribution of rehabilitation to motor and cognitive recovery cannot be isolated in a single patient report [3,14–16].

This case integrates several clinically relevant features, including hyperacute progression, extensive pansinusitis, anaerobic infection, associated venous sinus thrombosis, and documented rehabilitation outcomes. However, findings from a single case should be interpreted with caution. From a clinical perspective, early neuroimaging, including venous studies, is essential for diagnosis, prompt multidisciplinary management is critical for optimal outcomes, and rehabilitation should be considered an integral component of care rather than a secondary phase. [15,16].

### Strengths

This report provides a comprehensive multidisciplinary perspective, including surgical, microbiological, and rehabilitation aspects.

### Limitations

Limitations include the single-case design, limited generalizability, and absence of long-term follow-up data.

### Bias

Potential sources of bias include retrospective data collection and incomplete characterization of polymicrobial infection.

### Generalizability

Findings from this case should be interpreted cautiously, as variability in patient characteristics and treatment timing may influence outcomes.

## Conclusion

In conclusion, this case underscores the complexity of subdural empyema management and illustrates its clinical course and multidisciplinary management in the context of superior sagittal sinus thrombosis. However, conclusions regarding treatment effectiveness cannot be drawn from a single case. Future studies are warranted to better define prognostic factors and to standardize rehabilitation approaches in this patient population.

### Learning points

- Rapid progression of subdural empyema requires urgent recognition.
- Early neuroimaging should be considered in patients with headache and neurological symptoms suggestive of intracranial complications.
- Multidisciplinary management is recommended in current practice.
- Cerebral venous sinus thrombosis requires careful anticoagulation.
- Anaerobic pathogens like *Prevotella oris* should be considered.
- Structured neurorehabilitation may support functional recovery.

### Patient perspective

Patient emphasized the importance of early recognition of symptoms and multidisciplinary care. The patient reported progressive improvement in independence during the rehabilitation period.
